# Literature classification for semi-automated updating of biological knowledgebases

**DOI:** 10.1186/1471-2164-14-S5-S14

**Published:** 2013-10-16

**Authors:** Lars Rønn Olsen, Ulrich Johan Kudahl, Ole Winther, Vladimir Brusic

**Affiliations:** 1 Bioinformatics Centre, Department of Biology, University of Copenhagen, Copenhagen, Denmark; 2 Cancer Vaccine Center, Dana-Farber Cancer Institute, Boston, MA, USA; 3 Center for Biological Sequence Analysis, Department of Systems Biology, Technical University of Denmark, Lyngby, Denmark; 4 Cognitive Systems, DTU Compute, Technical University of Denmark, Lyngby, Denmark; 5 Department of Computer Science, Metropolitan College, Boston University, Boston MA, USA

**Keywords:** Text mining, machine learning, biological databases, automation

## Abstract

**Background:**

As the output of biological assays increase in resolution and volume, the body of specialized biological data, such as functional annotations of gene and protein sequences, enables extraction of higher-level knowledge needed for practical application in bioinformatics. Whereas common types of biological data, such as sequence data, are extensively stored in biological databases, functional annotations, such as immunological epitopes, are found primarily in semi-structured formats or free text embedded in primary scientific literature.

**Results:**

We defined and applied a machine learning approach for literature classification to support updating of TANTIGEN, a knowledgebase of tumor T-cell antigens. Abstracts from PubMed were downloaded and classified as either "relevant" or "irrelevant" for database update. Training and five-fold cross-validation of a *k*-NN classifier on 310 abstracts yielded classification accuracy of 0.95, thus showing significant value in support of data extraction from the literature.

**Conclusion:**

We here propose a conceptual framework for semi-automated extraction of epitope data embedded in scientific literature using principles from text mining and machine learning. The addition of such data will aid in the transition of biological databases to knowledgebases.

## Background

Databases are the cornerstone of bioinformatics analyses. Experimental methods keep advancing and high-throughput methods keep increasing in volume, the number of biological data repositories are growing rapidly [1]. Similarly, the quantity and complexity of the data are growing requiring both the refinement of analyses and higher resolution and accuracy of results. In addition to the most commonly used biological data types such as sequence data (gene and protein), structural data, and quantitative data (gene and protein expression), the increasing amount of high-level functional annotations of biological sequences are needed to enable detailed studies of biological systems. These high-level annotations are also captured in the databases, but to a much smaller degree than the essential data types. The literature, however, is a rich source of functional annotation information, and combining these two types of sources provides a body of data, information, and knowledge needed for practical application in bioinformatics and clinical bioinformatics. Extraction of knowledge from these sources is facilitated through emerging knowledgebases (KB) that enable not only data extraction, but also data mining, extraction of patterns hidden in the data, and predictive modeling. Thus, KB bring bioinformatics one step closer to the experimental setting compared to traditional databases since they are intended to enable summarization of hundreds of thousands of data points and *in silico *simulation of experiments all in one place.

A knowledge-based system (KBS) is a computational system that uses logic, statistics and artificial intelligence tools for support in decision making and solving complex problems. The KBS include specialist databases designed for data mining tasks and knowledge management databases (knowledgebases). A KBS is a system comprising a KB, a set of analytical tools, a logic unit, and user interface. The logic unit connects user queries and determines, using workflows, how analytical tools are applied to the knowledge base to perform the analysis and produce the results. Primary sources such as UniProt [2] or GenBank [3], as well as specialized databases such as The Influenza Research Database (IRD) [4] and the Los Alamos National Laboratory HIV Databases (http://www.hiv.lanl.gov/), offer a number of integrated tools and annotated data, but their analytical workflows are limited to basic operations. Examples of more advanced KBS include FlaviDb a KBS of flavivirus antigens, [5], FluKB a KBS of influenza antigens (http://research4.dfci.harvard.edu/cvc/flukb/), and TANTIGEN a KBS of tumor antigens (http://cvc.dfci.harvard.edu/tadb/index.html). KBS focus on a narrow domain, and a set of analytical tools to perform complex analyses and decision support. KBS must contain sufficient data, and annotations to enable data mining for summarization, pattern discovery and building of models that simulate behavior of real systems. For example FlaviDb, enables summarization of diversity of sequences for more than 50 species of flaviviruses. It also enables the analysis of the complete set of predicted T cell epitopes for 15 common HLA alleles and has the capacity to display the complete landscape of both predicted and experimentally verified HLA associated peptides. The extension of antigen analysis functionalities with FluKB enables analysis of cross-reactivity of all entries for neutralizing antibodies. Both these examples focus on identification, prediction, variability analysis and cross-reactivity of immune epitopes. The implementation of workflows in these KBS enables complex analyses to be performed by filling a single query form and results are presented in a single report.

To get high quality results, we must ensure that KBS are up to date and error-free (to the extent possible). Since the information in KBS is derived from multiple sources, providing high quality updates is complex. Manual updating of KBS is impractical, so automation of the updating process is needed. Automated updating of data and annotation by extracting data from primary databases such as UniProt, GenBank, or IEDB is relatively simple since these sources enable export of data using standardized formats, mainly XML. Ideally, functional annotations will be deposited by direct submission to appropriate databases by the discoverers, but a historical lack of submission standards for higher-level biological data, has lead to the vast majority of this information being recording only in primary scientific literature. The use of data embedded in primary scientific literature accessible through PubMed or Google Scholar, is markedly more complex. The information stored in abstracts or full texts is, at best, semi-structured, but typically it is provided as free text. Given that as many as tens of thousands of articles may be published each year on a given topic, access to this information and assessment of its relevance require efficient methods for identification of publications of interest and rapid assessment of their suitability for inclusion in the KBS. Such analysis is facilitated through use of text mining techniques, ranging from simple statistical pattern learning based on term frequencies, to complex natural language processing techniques in order to produce text categorization, document summarization, information retrieval, and ultimately the data mining [6]. A long-term solution for this issue invariably involves standardizing submission and storage of complex biological data, but the knowledge currently embedded in the literature remains available for extraction. Text mining operations have previously been applied for specific knowledge extraction for vaccine development [7], as well as document classification for separation of abstracts by topic [8] and for semi-automated extraction of allergen cross-reactivity information [9]. In this article, we will define the conceptual framework for semi-automated updating of our tumor antigen knowledgebase, TANTIGEN, using data parsing, basic text mining operations, and a standardized submission system.

## Results and discussion

### Conceptual framework

Depending on the content of the KBS one wishes to update, there are issues pertaining to the complexity of biological data that require considerations. Particularly we must address the diversity of data types, diversity of data formats, dispersion of data across different sources, and size of data sets. There are many biological data types - the most common include sequence data (nucleotide or protein), molecular structures, expression data, and functional annotations. Data can be stored and retrieved either as structured text, table formats, semantic web formats (such as RDP, OWL, or XML), or non-structured text. Depending on the target data format, retrieval can be performed by direct extraction, parsing, text mining, or manual extraction. Text mining, manual extraction, or a combination of these two is common in extracting the high-level data, such as functional annotations. Data availability and individual entry size vary between different data types, presenting a computational challenge in terms of retrieval, handling, analysis, and storage. Additional factors that affect the complexity of the updating task are data heterogeneity, integration of multiple data types after retrieval, as well as provenance tracking for quality assessment [10].

**Figure 1:**
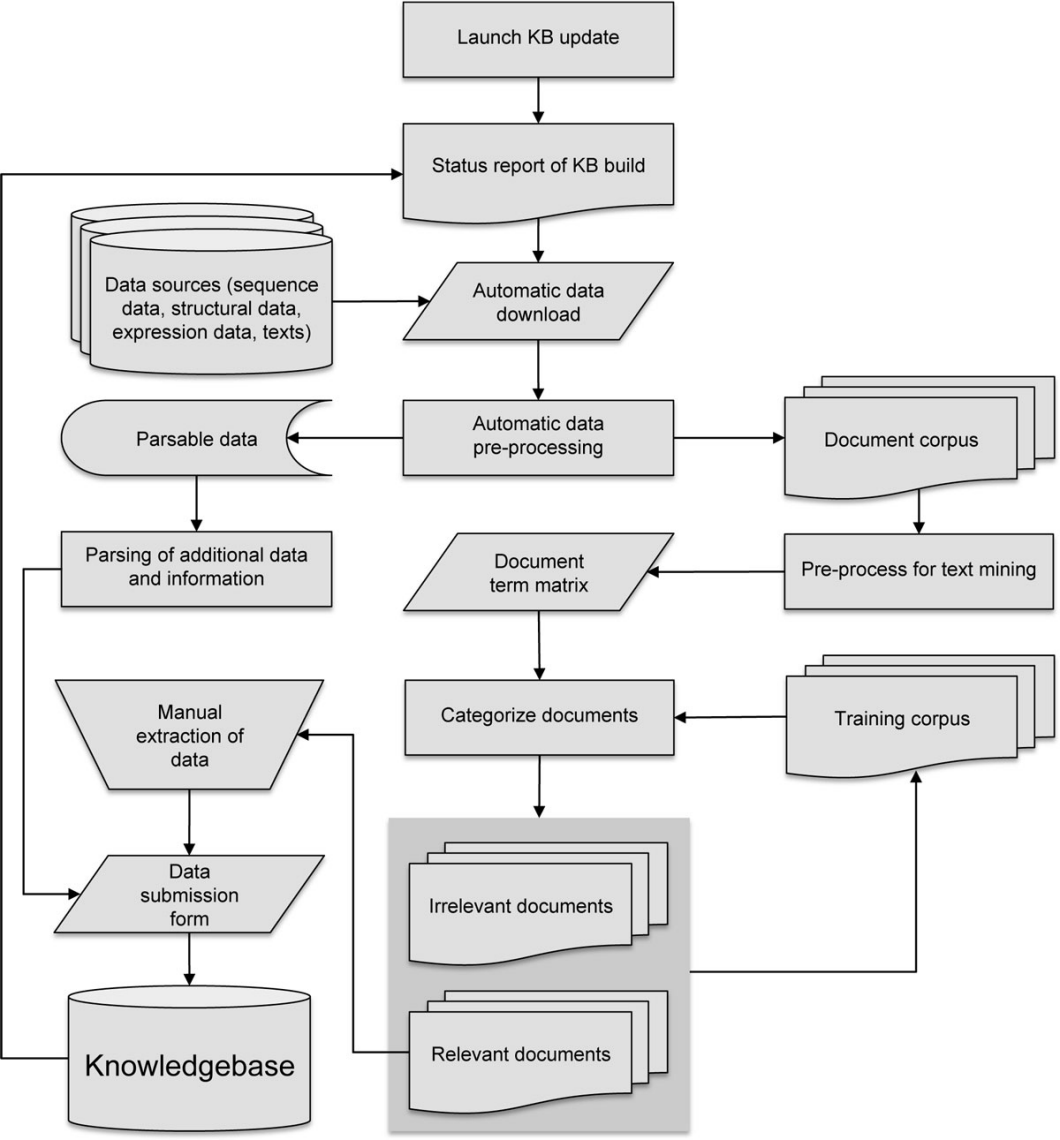
**Flow chart of tasks in conceptual framework for semi-automated updating of knowledgebases**.

To address these issues we have formalized a number of common tasks pertaining to knowledgebase updating into a conceptual framework for updating biological KBS, shown in Figure 1.

Step 1: *Produce status report of current knowledgebase build*. This report will serve as the filter for the two main updating tasks: update of existing entries and update of data body by introduction of new entries.

Step 2: *Automatic download of data from selected sources*. Most biological data repositories enable full download of latest database build and most allow automated retrieval via GNU Wget or FTP clients. If automatic download is not possible, this step can be performed manually.

Step 3: *Automatic data pre-processing*. Depending on the data format, pre-processing steps can be automated in various ways. For simple syntax-based formats such as XML, parsing of desired data is possible, where for non-standardized formats, such as raw text, pre-processing involves tasks derived from text mining, such as word stemming, stop word removal, and generation of document-term matrix (DTM) [11].

Step 4: *Text categorization*. If the desired information is not available in a standardized format - for example that it is only available in primary scientific literature, the text mining or machine learning methods can be applied to direct and streamline the manual extraction. A text corpus may contain documents that fall into two or more categories, of which only one or a few are of interest for a given task. To maximize the efficiency of manual data extraction, it is helpful to classify documents before embarking on data extraction. Options for classification using machine learning methods include: unsupervised methods such as clustering and blind signal separation, or supervised methods such as artificial neural networks, support vector machines, nearest neighbor methods, Naive Bayes, decision trees, among others [6]. For some of these algorithms, feature extraction using matrix factorization methods, such as principal component analysis (singular value decompression) can be useful to reduce dimensionality of DTM, which can become quite large.

Step 5: *Manually extract data and information from categorized texts*. Some higher-level data types, such as functional annotations, are often found in tables, figures, legends, or supplementary materials of primary scientific articles, making automated extraction of this information highly complex or practically impossible [7]. A manual extraction step may therefore be needed and simultaneously allow for quality control.

Step 6: *Submission of new or updated entries to the KBS*. Submission of extracted data to the KBS should be standardized to the highest degree possible in order to ensure the adherence to standardized format and quality of an entry. The use of a standardized submission form allows non-experts to perform the task of updating. Automated extraction of related data from primary databases can minimize the manual entry of data and mismatches between existing entries addition to entries, provide automated error detection to be manually addressed.

Step 7: *Refining categorization by increasing the training corpus*. Each manually inspected document (classified either as relevant or irrelevant) represents a new addition to the training data used for documentation categorization. In addition to refining the model and improving performance, a feedback loop to the classification module reduces the need for a large initial training corpus.

### Case study: TANTIGEN tumor T cell antigen database

Selection of useful tumor T cell antigens represents a major bottleneck to the study and design of cancer immunotherapies. The methods of selecting immunotherapy targets involve the selection of antigens and the analysis of their immune epitopes. This process has been greatly enhanced by the use of computational immunology methods [12]. However, as computational efforts produce vast amounts of potential targets, the bottleneck is shifted to the wet lab, where the vaccine target candidates must be validated for both relevance and immunogenicity before they are included in potential vaccine constructs. Great advances have been made in techniques for high-throughput epitope validation [13,14], but as computational methods grow ever more powerful, so does the need for post-analysis verification of results. Efficient cataloguing of experimentally validated epitopes for cross-referencing of new predictions with past experimental data is a valuable resource that could reduce the need for and streamline further experimentation. Several specialized resources for this and similar purposes have been established, for example: IRD [4], The HIV databases (http://www.hiv.lanl.gov), Human Papillomavirus T cell Antigen Database for HPV (http://cvc.dfci.harvard.edu/hpv/index.html), as well as general HLA binder repositories such as SYFPEITHI [15] and the Immune Epitope Database (IEDB) [16].

The TANTIGEN database was established in 2007 as a tumor-specific T cell antigen database. It provides the scientific community with a curated repository of experimentally validated tumor T-cell antigens, and matched T-cell epitopes and HLA binders. Each antigen entry contains detailed information about somatic mutations from the Catalogue of Somatic Mutations in Cancer (COSMIC) [17], splice isoforms from UniProt/Swiss-Prot, gene expression profiles from UniGene, and known T-cell epitopes from secondary databases or literature. Additionally, TANTIGEN is equipped with a number of analysis tools such as BLAST search [18], multiple sequence alignment using MAFFT [19], T-cell epitope/HLA ligand prediction [20,21] and visualization, and tumor antigen classification [22].

### Updating TANTIGEN

Keeping up-to-date data in a KBS represents a major bottleneck in the maintenance of TANTIGEN. In 2012, 7,322 articles responding to the keywords "tumor antigen" were indexed in PubMed. Although many of these articles may not contain tumor T cell antigens, the growing quantities of literature represents a major bottleneck in the maintenance of curated databases [7].

The data types to be updated in TANTIGEN are experimentally characterized T cell epitopes and HLA ligands, and expression and variability information for the proteins that harbor them. In build 1 of TANTIGEN, these data were collected from six different sources: manual collection from the literature, the Peptide database: T cell-defined tumor antigens (http://www.cancerimmunity.org/peptide/), the listing of human tumor antigens recognized by T cells by Parmiani and colleagues [23,24], and parsing from IEDB, as well as four other public databases that are outdated or unavailable at present. The primary resource for these data remains manual collection from the literature, as no primary database is actively collecting or curating tumor antigen data. IEDB offers some curated cancer data (2.7% of available data curated as of November 2009 [16]), but in their February 2011 newsletter they announced that they will no longer curate cancer tumor epitope data.

#### Preliminary filtering of literature

A simple keyword search for the terms "cancer OR tumor OR antigen OR epitope" in PubMed, yielded >552,000 results (from December 1, 2009 - March 29 2013). When keyword stringency increased the number of useful publications decreased to a workable level (Table 1). For this task, we decided to use the search term "(tumor OR cancer) AND (antigen OR epitope)", which yields 48,130 hits in PubMed. Keyword search terms could be further expanded or refined, by reiterating either manually or using feature extraction of discriminative terms using machine-learning methods. Manually sorting of these articles is extremely laborious task. PubMed is currently growing at approximately 4% per year [25], so the issue will only increase. It is therefore advantageous to automate the classification of publication content before manually extracting relevant information. For this task, we employed an adapted version of the conceptual framework to update TANTIGEN.

**Table 1:** Examples of PubMed results from a selection of keyword searches (publication data from December 1, 2009 - March 29, 2013).

Keyword	PubMed hits
cancer OR tumor OR antigen OR epitope	552309

(tumor OR cancer) AND (antigen OR epitope)	45517

tumor AND antigen	40525

tumor antigen	22264

tumor AND antigen AND epitope	3057

tumor AND antigen AND epitope AND T cell	852

"tumor antigen"	642

#### Formal approach to updating

Step 1: *Status report of TANTIGEN build 1*. The status report for TANTIGEN lists 251 unique proteins and corresponding UniProt accession numbers. Many of these proteins have multiple splice isoforms for which UniProt accession numbers are also listed. All UniProt accession numbers are listed as these entries are subject to updating by direct parsing from UniProt data downloads. Similarly, PubMed IDs are listed for all referenced articles. These articles represent relevant literature and corresponding abstracts can be directly parsed from the PubMed abstract download to the training document set. The build 1 of TANTIGEN has 4,006 curated antigen entries.

Step 2: *Automatic data download*. The latest versions of UniProt and COSMIC are downloadable as XML files from the database web sites. PubMed results can be narrowed down by search term, in this case we used "(cancer OR tumor) AND (antigen OR epitope)", but this can be refined in later iterations if suitable. Due to the very high volume of abstracts in PubMed, query results can also be filtered by date, and we here filtered out articles published before the last TANTIGEN update. Search results are downloadable in XML format.

Step 3: *Automatic data pre-processing*. The COSMIC and UniProt XML downloads needed no further pre-processing for parsing. The PubMed abstracts were extracted from the XML and parsed into a text corpus format for pre-processing. The following tasks were performed on the corpus: lower case transformation, removal of stop words, removal of general punctuation, word stemming, and white space stripping. The numbers are usually removed in text mining preprocessing, but it was not done here because we needed to preserve the terms defining HLA alleles, CD receptors, and other immunologically relevant descriptors.

Step 4: *Abstract categorization*. The resulting DTM was Tf-Idf transformed, and each abstract was classified using a *k*-Nearest Neighbor (*k*-NN) classifier trained on 226 manually pre-classified abstracts. Iterative refinement of the algorithm showed that a six nearest neighbors model yielded the best results. Each abstract in the corpus was given a probability score based on the ratio of relevant neighbors in the model. The output list was ordered from most probable to least, thus eliminating the need to define a static threshold.

Step 5: *Manually extract antigen data from literature*. The articles corresponding to each abstract classified as relevant were accessed through PubMed or publishing journal. Epitopes, HLA ligands and related data, such as HLA restriction and protein of origin, were extracted. For TANTIGEN build 2, we manually searched the top 273 articles out of classified 48,130 articles. The cutoff of 273 articles was chosen when article relevance started decreasing drastically in the ordered list during manual data extraction.

Step 6: *Submission of data*. Submission was done by filling out a standardized TANTIGEN submission form for each antigen. Additional information was parsed directly from the downloaded UniProt XML, based on the protein of origin. Similarly, mutation entries and splice variants were automatically linked by cross-referencing with COSMIC XML. Entries in TANTIGEN were automatically linked to each other where applicable (splice isoforms, mutation entries, etc.). Updating of existing entries was performed by automated parsing form UniProt XML, as some entries were removed, assigned new accession, updated with more splice isoforms. This step also serves as a error detection: if an existing entry in TANTIGEN does not match the information entered in the standardized submission form, the user is notified and prompted to determine whether the existing entry, the submission, or both are erroneous. Similarly, if protein information extracted from UniProt does not match that in COSMIC, the user will be prompted to resolve the issue, thus increasing data quality.

Step 7: *Refine training set with new entries*. The TANTIGEN submission form has an addition field, where the curator performing the manual submission is prompted to classify the article as "relevant" or "irrelevant". This feature was used to feed manually inspected abstracts back into the training corpus, to increase its size and thus performance. The false positives and false negatives were fed back, but only a randomly selected fraction of true positives and true negatives were fed back into the training corpus, as these may further bias a potentially already biased model.

### Results of TANTIGEN update

#### Accuracy of classification

The average accuracy in the five-fold cross-validation training of the *k*-NN model with 6 nearest neighbors was 0.95 with sensitivity of 0.96 and specificity of 0.93. Model performance is likely to increase with the increase of training set size, and particularly the addition of false positives from the manual extraction step. True positive should also be added to the training corpus, but including *all *true positives may further bias a potentially biased model. Special care should be taken in initial classification rounds to extract and include false negatives, as low sensitivity is highly detrimental to the quality and completeness of the update. Wrongfully discarding relevant literature will not only lead to, potentially permanent, loss of valuable data, but also negatively affect classifier performance, when misclassified training data is fed back into the model.

#### Results of manual extraction of tumor T-cell antigens

Manual extraction of new antigenic proteins and tumor T-cell antigens was performed from the classified literature. Since classification was based on the six nearest neighbors, the body of classified abstracts was divided in seven groups, corresponding to whether an abstract had from zero to six relevant neighbors in the training set. Out of the 48,130 classified abstracts, 117 had six relevant neighbors, 156 had five, 212 had four, 859 had three, 3,489 had two, 12,738 had one, and 30,856 abstracts had zero relevant neighbors. We manually examined the top 273 scoring papers in which we found 13 new antigenic proteins harboring 32 new tumor T-cell epitopes. Additionally, we found more than 100 new T-cell epitopes discovered in proteins already recorded as tumor antigens in TANTIGEN.

#### Training set refinement iteratively increase classification accuracy

**Figure 2:**
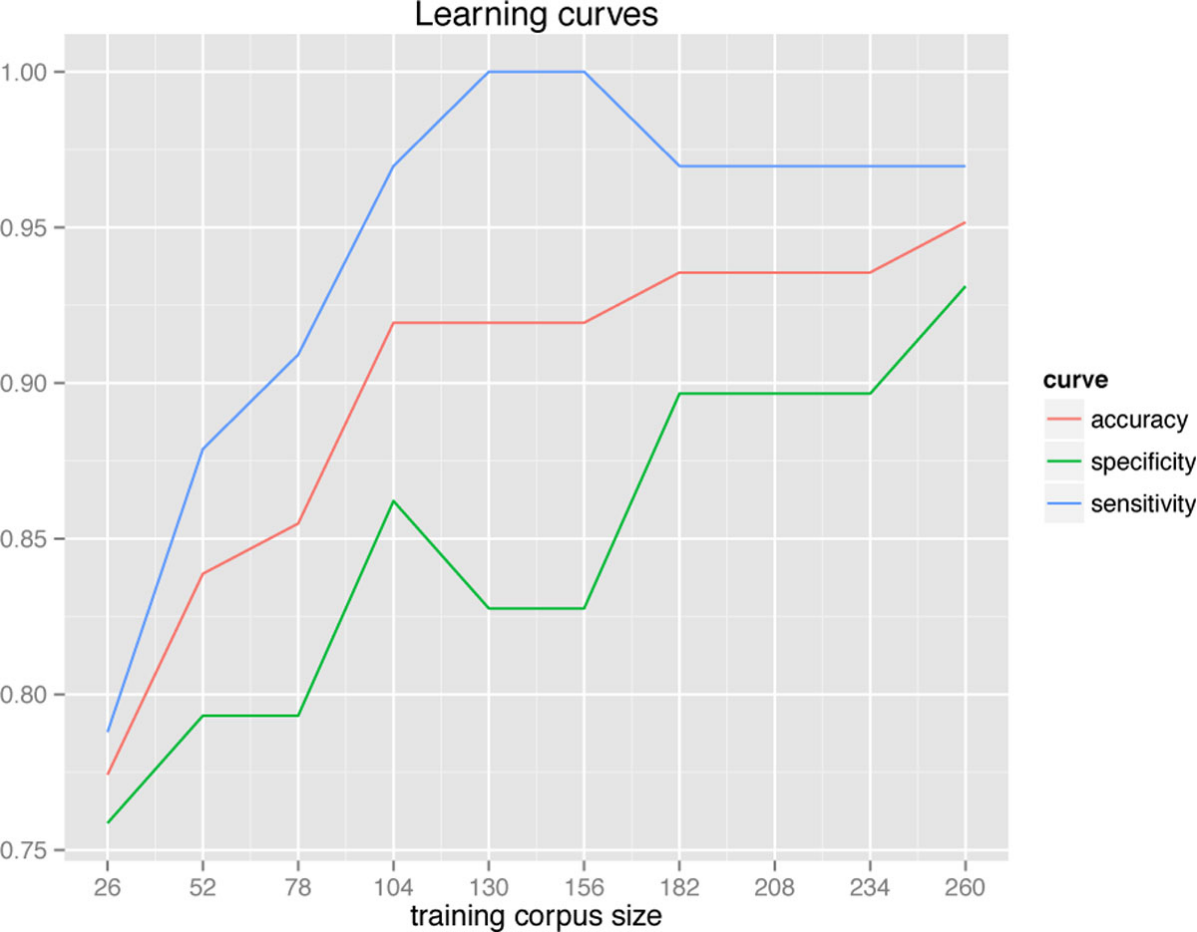
**Learning curve for training sets of increasing size**. Initial training set consisted of 13 relevant and 13 irrelevant abstracts. Training set was increased to 260 abstracts in increments of 26 additional abstracts. Test set was fixed at 50 abstracts, 25 relevant and 25 irrelevant.

The performance of the document classification model is expected to gradually increase as the size of the training corpus is increased with each database update. Learning curves for accuracy, sensitivity, and specificity constructed by gradually increasing the training corpus for a test corpus fixed to 50 abstracts (25 relevant and irrelevant, respectively) supports this notion (Figure 2). Although the sensitivity and specificity show some fluctuations, accuracy is observed to steadily increase as the training set size is increased in increments of 26 abstracts (13 relevant and irrelevant, respectively). The learning curves will likely plateau with the addition of further training abstracts, although any increase in sensitivity will add to data completeness, and increased specificity will minimize labor intensity.

#### Abstract category signatures

**Figure 3:**
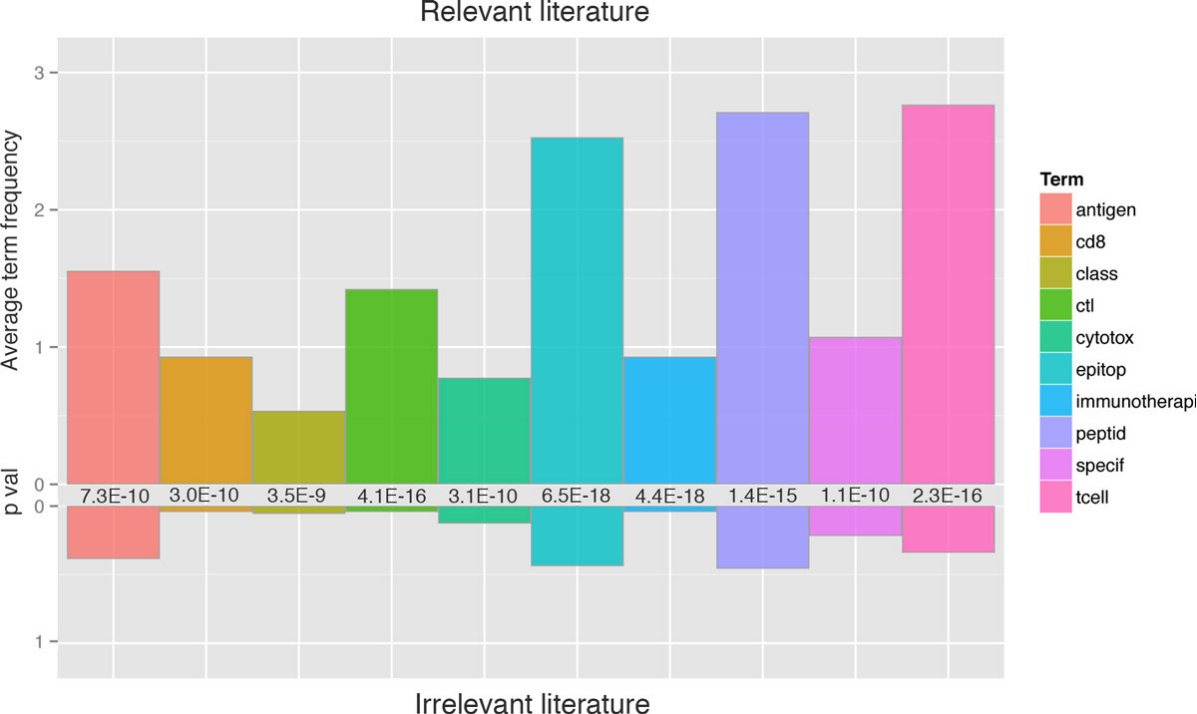
**Average frequency of the top ten most discriminative terms between relevant (above x axis) and irrelevant abstracts (below x axis)**. Significance of difference is based on t test of term frequency between corpora and p-values are listed between bars. Terms are stemmed to ensure completeness in term count.

The DTM of the training corpus contains more than 5,600 terms. Most are very rare terms present in only one or a few abstracts, and have very little influence on abstract classification as corresponding to either relevant or irrelevant articles. Rare terms can be removed by setting a sparsity threshold if DTM dimensions become too large. Examining the top ten terms, most discriminative between abstracts of relevant and irrelevant articles (determined by t test), show a distinct signature and reveal particular emphasis on such terms as "immunotherapy", "epitope", "T cell", and "CTL" (Figure 3). These terms are likely the main drivers of classification and may very well be sufficient to support the main task of classification. Notable is the fact that all discriminating terms are predominant in relevant abstracts, which may explain that sensitivity of classification is higher than specificity. This is most likely due to the highly specific nature of the relevant abstracts, whereas irrelevant abstracts are a much broader class. However, these terms are still represented in the corpus of irrelevant literature, so a machine learning approach to classification is highly likely to outperform a simple keyword search.

## Conclusion

Specialized biological databases are gradually moving from data repositories towards knowledge-based systems. Enriching basic biological data with higher-level functional annotations and facilitating specialized analyses in organized workflows enables extraction of higher-level knowledge. Currently, however, functional annotations are primarily stored in the literature, rather than in standardized formats of primary biological databases. As the quantity of this information increases, easy access to multiple layers of biological data and information enables improved extraction of knowledge, thus increasing the value to the user.

We here present a conceptual framework for automating the process of updating biological databases and knowledgebases with standardized non-standardized data from both primary and secondary data repositories, as well as literature. We deployed a text mining-based approach to categorize literature, based on defining term signatures of freely available article abstracts, which enable significantly faster manual extraction of relevant data. We have applied this conceptual framework to literature for updating the TANTIGEN KBS of tumor T cell antigens. Training of a *k*-NN classifier on 260 abstracts yielded classification accuracy of 0.95, thus showing significant value in support of data extraction from the literature.

## Methods

### Data sources

**Figure 4:**
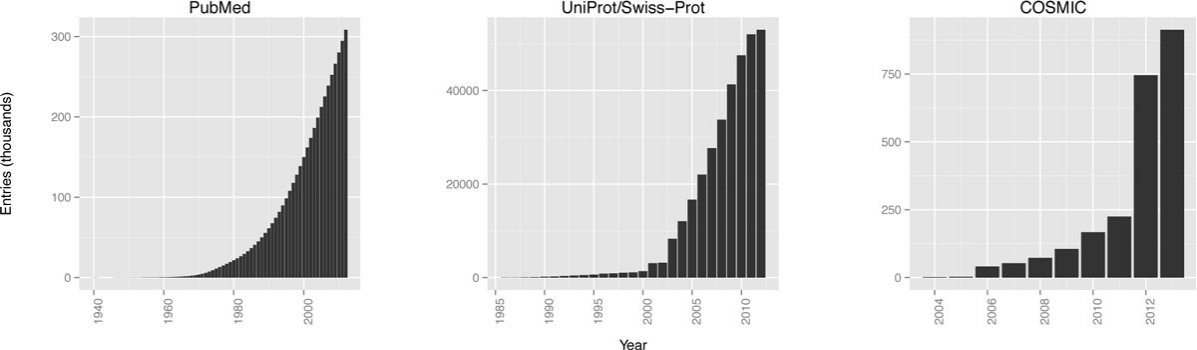
**Number of entries in PubMed, UniProt/Swiss-Prot, and COSMIC**. Entries in PubMed were filtered by the search term *"(tumor OR cancer) AND (antigen OR epitope)"*.

Data for updating TANTIGEN were extracted from three primary databases: UniProt/Swiss-Prot for protein data and information, COSMIC for data about somatic mutations, and PubMed for published literature about tumor antigens. All three databases are extensive repositories for their respective data types, and the quantity of data is increasing steadily (Figure 4).

All three databases offer download in XML format, where the desired information was directly parsable from UniProt/Swiss-Prot and COSMIC, but only abstracts were available for PubMed entries and protein information and epitopes from these entries required manual extraction. To aid the process of KB update, text mining tools and machine learning tools were employed to filter text entries as either relevant (containing T cell epitopes) or irrelevant (not containing T cell epitopes).

### Classification of literature abstracts

#### Corpus

A corpus for classification was extracted from PubMed using the search terms *"(tumor OR cancer) AND (antigen OR epitope)"*. Each entry in the corpus contains the article abstract, the titles, and the MeSH terms. Before classification, a number of term transformation steps were taken: lower case transformation, removal of numbers, removal of stop words, removal of punctuation, word stemming, synonym consolidation using the WordNet database [26], and white space removal. Term pre-processing of text corpus was done using the R package *tm *[27,28]. After term counting, term frequency-inverse document frequency (Tf-Idf) transformation was applied for background correction [29].

#### Classification

Abstracts were classified using the *k*-NN algorithm [30] from the R package *class*. The classifier was trained and performance evaluated for 1-155 nearest neighbors using five-fold cross-validation on a set of 310 abstracts (155 abstracts of irrelevant articles and 155 abstracts of relevant articles). This training set was manually assembled for initial training. Classification was done based on 6 neighbors in the *k*-NN algorithm, since this number of neighbors proved most accurate.

#### Abstract category signatures

A signature of the top ten terms most discriminating between relevant and irrelevant literature was extracted by t-test of differential term occurrence in relevant and irrelevant abstracts. The average term count was calculated for the ten most discriminating terms, i.e. the terms with the lowest p-values.

## Availability of supporting data

The TANTIGEN training corpus is available in raw form at http://cvc.dfci.harvard.edu/tadb/download/

## List of abbreviations used

KB: Knowledgebase; Tf-Idf: Term frequency-inverse document frequency; DTM: Document-term matrix; *k*-NN: *k*-Nearest Neighbor algorithm; XML: Extensible Markup Language.

## Competing interests

The authors declare that they have no competing interests.

## Authors' contributions

LRO: Conceived of framework and case study, performed case study, prepared manuscript. UJK: Contributed to conceiving the case study, contributed to case study (particularly data handling, data retrieval), critically reviewed manuscript. OW: Contributed to case study (particularly text mining tasks), critically reviewed manuscript. VB: Conceived of framework and case study, prepared manuscript.

## Declaration

Publication of this article was funded by the Novo Nordisk Foundation.

This article has been published as part of *BMC Genomics *Volume 14 Supplement 5, 2013: Twelfth International Conference on Bioinformatics (InCoB2013): Computational biology. The full contents of the supplement are available online at http://www.biomedcentral.com/bmcgenomics/supplements/14/S5.
